# Predictors for affected stroke territory and outcome of acute stroke treatments are different for posterior versus anterior circulation stroke

**DOI:** 10.1038/s41598-021-89871-4

**Published:** 2021-05-18

**Authors:** H. Handelsmann, L. Herzog, Z. Kulcsar, A. R. Luft, S. Wegener

**Affiliations:** 1 Department of Neurology and Clinical Neuroscience Center, University Hospital Zurich and University of Zurich, Frauenklinikstrasse 26, 8091 Zurich, Switzerland; 2 Epidemiology, Biostatistics and Prevention Institute (EBPI), University of Zurich, Zurich, Switzerland; 3 Institute of Data Analysis and Process Design, ZHAW Winterthur, Winterthur, Switzerland; 4 Department of Neuroradiology and Clinical Neuroscience Center, University Hospital Zurich and University of Zurich, Zurich, Switzerland; 5 Cereneo Center for Neurology and Rehabilitation, Vitznau, Switzerland

**Keywords:** Neurology, Risk factors

## Abstract

Distinct patient characteristics have been proposed for ischaemic stroke in the anterior versus posterior circulation. However, data on functional outcome according to stroke territory in patients with acute stroke treatment are conflicting and information on outcome predictors is scarce. In this retrospective study, we analysed functional outcome in 517 patients with stroke and thrombolysis and/or thrombectomy treated at the University Hospital Zurich. We compared clinical factors and performed multivariate logistic regression analyses investigating the effect of outcome predictors according to stroke territory. Of the 517 patients included, 80 (15.5%) suffered a posterior circulation stroke (PCS). PCS patients were less often female (32.5% vs. 45.5%, *p* = *0.031*), received thrombectomy less often (28.7% vs. 48.3%, *p* = *0.001*), and had lower median admission NIHSS scores (5 vs. 10, *p* < *0.001*) as well as a better median three months functional outcome (mRS 1 vs. 2, *p* = *0.010*). Predictors for functional outcome were admission NIHSS (OR 0.864, 95% CI 0.790–0.944, *p* = *0.001*) in PCS and age (OR 0.952, 95% CI 0.935–0.970, *p* < *0.001*), known symptom onset (OR 1.869, 95% CI 1.111–3.144, *p* = *0.018*) and admission NIHSS (OR 0.840, 95% CI 0.806–0.876, *p* < *0.001*) in ACS. Acutely treated PCS and ACS patients differed in their baseline and treatment characteristics. We identified specific functional outcome predictors of thrombolysis and/or thrombectomy success for each stroke territory.

## Introduction

Stroke is the second leading cause of death worldwide^1^, with 80–87% of strokes being ischaemic^2–4^. Ischaemic strokes can be further specified according to their vascular territory as anterior (ACS, from internal carotid arteries) and posterior circulation ischaemic stroke (PCS, from vertebral arteries). About 20% of the cerebral blood flow is directed through the posterior cerebral circulation, resulting in approximately one fifth of ischaemic strokes being PCS^2^. Usually PCS and ACS are associated with clinically distinct symptoms^5^. This is intuitively clear since different brain regions are affected. However, in addition, several previous studies comparing PCS and ACS have proposed different patient characteristics regarding demographics, cardiovascular risk factors and stroke aetiology specific for the stroke territory^5–9^. Overall, patients suffering a posterior circulation ischaemic stroke tend to be younger^6–8,10^ and more often male compared to ACS patients^5–8,11^. Diabetes was more frequent in PCS, whereas atrial fibrillation was more common in ACS^6–8,11^. Stroke aetiologies have been reported to be unevenly distributed in PCS and ACS, though results vary a lot between studies^5,8,9^. The reasons for these differences remain elusive.

Acute ischaemic stroke can be treated with thrombolysis and thrombectomy to restore perfusion of the brain. Both methods are safe and effective in ACS^12^ as well as in PCS^13–16^. However, the success of both treatments depends on patient factors, stroke severity as well as time since symptom onset^17^. Yet, the National Institutes of Health Stroke Scale (NIHSS) which is commonly used to quantify stroke severity, incompletely depicts stroke symptoms of the posterior circulation^18^. Therefore, this scale may not be a suited tool to judge symptom severity or predict treatment success. The modified Rankin Scale (mRS) has been established to evaluate the degree of disability and functional outcome after stroke^19,20^. Comparisons of functional outcome after acute stroke treatment between PCS and ACS yielded conflicting results, with most studies proposing similar outcomes for both stroke territories^5,13,14,21–24^ and some finding a higher rate of disability in either PCS^8^ or ACS^25^. Since patients with PCS seem to be different from those with ACS, this implies that outcome may be determined by different factors for these vascular territories. However, information on specific outcome predictors according to stroke territory is scarce^14,15,21^.

The aim of this analysis was to search for different patient profiles in patients with PCS versus ACS subjected to thrombolysis and/or thrombectomy and therefore deemed significantly affected in an early time window. One goal was to identify specific predictors for the affected vascular territory by stroke. Additionally, we wanted to investigate if specific outcome predictors exist for the different vascular stroke territories.

## Results

### Patient characteristics

3594 patients with suspected stroke treated between 2014 and 2017 were screened from the Swiss Stroke Registry at the University Hospital of Zurich. Out of these patients, 583 fulfilled the inclusion criteria of ischaemic stroke treated with thrombolysis and/or thrombectomy and either written informed consent or no objection to data research according to the ethics protocol (KEH-ZH-Nr. 2014-0304). 21 patients were excluded due to missing information on three months mRS, another 26 patients due to concurrent stroke in both the posterior and anterior cerebral territory, and 19 patients due to stroke treatment after > 24 h (n = 13) or in-hospital events (n = 6). In the end, 517 patients were included in this data analysis. Out of the 517 patients, 80 (15.5%) had PCS and 437 (84.5%) had ACS.

Demographic data and information on the patients’ medical history are shown in Table 1. There was no difference in age between PCS and ACS patients (69.4 years, IQR 24 vs. 72.3 years, IQR 20, *p* = *0.187*). The proportion of female patients was significantly lower in PCS compared to ACS (32.5% versus 45.5%, *p* = *0.031*). PCS patients less often suffered from diabetes (5.0% vs. 14.4%, *p* = *0.021*) and atrial fibrillation (16.3% vs. 29.7%, *p* = *0.013*). Other cardiovascular risk factors including previous cardiovascular events as well as pre-stroke medication did not differ between the two groups.

**Table 1:** Patient clinical characteristics.

	All n = 517 (%)	ACS n = 437 (%)	PCS n = 80 (%)	P value
**Demographic data**				
Age, median years (IQR)	71.9 (20)	72.3 (20)	69.4 (24)	0.187
Female, n (%)	225 (43.5)	199 (45.5)	26 (32.5)	**0.031**
**Medical history and risk factors, n (%)**				
Stroke and/or TIA	70 (13.5)	57 (13.0)	13 (16.3)	0.441
Intracranial haemorrhage	6 (1.2)	6 (1.4)	0 (0)	0.597
Hypertension	349 (67.5)	299 (68.4)	50 (62.5)	0.299
Diabetes	67 (13.0)	63 (14.4)	4 (5.0)	**0.021**
Dyslipidaemia	292 (56.5)	248 (56.8)	44 (55.0)	0.772
Smoking	108 (20.9)	87 (19.9)	21 (26.3)	0.200
Atrial fibrillation	143 (27.7)	130 (29.7)	13 (16.3)	**0.013**
Coronary heart disease	95 (18.4)	82 (18.8)	13 (16.3)	0.593
Prosthetic valve	18 (3.5) n = 515	16 (3.7) n = 435	2 (2.5) n = 80	1.000
Low ejection fraction	10 (1.9) n = 513	9 (2.1) n = 435	1 (1.3) n = 79	1.000
Peripheral artery disease	31 (6.0)	26 (5.9)	5 (6.3)	0.803
**Pre-stroke medication, n (%)**				
Aspirin	157 (30.4) n = 516	134 (30.7) n = 436	23 (28.7) n = 80	0.723
Clopidogrel	33 (6.4) n = 514	26 (5.9) n = 434	7 (8.8) n = 80	0.355
Prasugrel	2 (0.4)	2 (0.5)	0 (0)	1.000
Ticagrelor	2 (0.4) n = 514	2 (0.5) n = 435	0 (0) n = 79	1.000
Dipyridamole	5 (1.0) n = 514	5 (1.1) n = 435	0 (0) n = 79	1.000
Vitamin K Antagonists	30 (5.8) n = 516	27 (6.2) n = 436	3 (3.8) n = 80	0.602
Heparin parenteral	3 (0.6) n = 516	3 (0.7) n = 436	0 (0) n = 80	1.000
DOACs	12 (2.3) n = 512	11 (2.5) n = 434	1 (1.3) n = 78	1.000
Antihypertensives	278 (53.8)	242 (55.4)	36 (45.0)	0.087
Lipid-lowering drugs	135 (26.2) n = 515	115 (26.4) n = 435	20 (25.0) n = 80	0.788

Clinical characteristics of all 517 patients and patient groups according to stroke territory, anterior circulation stroke (ACS) and posterior circulation stroke (PCS). Data are expressed as number of patients (n) and percentages or median and interquartile range (IQR). TIA indicates transient ischaemic attack; DOACs, direct oral anticoagulants (i.e. Rivaroxaban, Dabigatran, Apixaban). P values were obtained according to Mann–Whitney U test, Pearson’s Chi-squared test and Fisher’s Exact test, where appropriate. P values < 0.05 are shown in bold.

Stroke aetiologies were similar in both patient groups. The type of acute stroke treatment was significantly different between PCS and ACS (*p* = *0.005*). As expected, PCS patients less often received mechanical thrombectomy (28.7% vs. 48.3%, *p* = *0.001*). Treatment times including onset-to-door time (ODT), onset-to-treatment time (OTT) or last-time-seen-normal-to-door and last-time-seen-normal-to-treatment time, for unknown symptom onset respectively, as well as the door-to-treatment time (DTT) were similar for both stroke territories except for patients with known symptom onset who were treated with intravenous thrombolysis. In this subgroup, PCS patients had a longer OTT compared to ACS patients (median time 151.5 min, IQR 98 vs. 128 min, IQR 68, *p* = *0.014*) (Table 2).

**Table 2:** Stroke aetiology and acute treatment information.

	All n = 517 (%)	ACS n = 437 (%)	PCS n = 80 (%)	P value
**Stroke aetiology, n (%)**				0.229
Large artery atherosclerosis	72 (13.9)	58 (13.3)	14 (17.5)	
Cardiac	189 (36.6)	169 (38.7)	20 (25.0)	
Lacunar	17 (3.3)	14 (3.2)	3 (3.8)	
Dissection	27 (5.2)	22 (5.0)	5 (6.3)	
Rare or multiple causes	18 (3.5)	14 (3.2)	4 (5.0)	
Unknown cause	194 (37.5)	160 (36.6)	34 (42.5)	
**Acute stroke treatment, n (%)**				**0.005**
Only IVT	283 (54.7)	226 (51.7)	57 (71.3)	
Only IAT	75 (14.5)	67 (15.3)	8 (10.0)	
Both IVT and IAT	159 (30.8)	144 (33.0)	15 (18.8)	
IAT (with or without IVT)	234 (45.3)	211 (48.3)	23 (28.7)	**0.001**
**Treatment times, median minutes (IQR)**				
Known time of symptom onset, n (%)	397 (76.8)	331 (75.7)	66 (82.5)	0.188
Onset-to-door*	95 (105) n = 396	90 (97) n = 331	114 (130) n = 65	0.071
Onset-to-treatment*	137 (82) n = 397	135 (79) n = 331	155 (98) n = 66	0.069
All IVT (onset-to-IVT)*	130 (71) n = 365	128 (68) n = 301	151.5 (98) n = 64	**0.014**
Only IAT (onset-to-groin)*	340 (228) n = 32	315 (221) n = 30	710 n = 2	0.465
Last time seen normal-to-door**	330 (365) n = 119	335 (354) n = 106	230 (541) n = 13	0.578
Last time seen normal-to-treatment**	420 (452) n = 120	420 (435) n = 106	375 (633) n = 14	0.879
All IVT (last time seen normal-to-IVT)**	300 (338) n = 77	330 (345) n = 69	237.5 (251) n = 8	0.279
Only IAT (last time seen normal-to-groin)**	695 (520) n = 43	695 (523) n = 37	615 (698) n = 6	0.798
Door-to-treatment	43 (50) n = 515	44 (49) n = 437	41.5 (68) n = 78	0.726
All IVT (door-to-IVT)	37 (37) n = 440	36.5 (34) n = 370	37.5 (59) n = 70	0.995
Only IAT (door-to-groin)	180 (115) n = 75	180 (110) n = 67	127.5 (146) n = 8	0.799

Information on stroke aetiology and acute stroke treatment of all 517 patients and patient groups according to stroke territory, ACS and PCS.

*Marked variables only contain patients with known symptom onset versus **unknown symptom onset. Data are expressed as number of patients (n) and percentages or median and interquartile range (IQR). IVT indicates intravenous thrombolysis; IAT, intra-arterial therapy. P values were obtained according to Mann–Whitney U test, Pearson’s Chi-squared test and Fisher’s Exact test, where appropriate. P values < 0.05 are shown in bold.

There were no differences regarding vital parameters on admission between PCS and ACS. PCS patients presented with a lower NIHSS on admission (median NIHSS 5, IQR 8 vs. 10, IQR 11, *p* < *0.001*) and had a lower NIHSS after 24 h (median NIHSS 2, IQR 5 vs. 5, IQR 9, *p* < *0.001*). The pre-stroke modified Rankin Scale was similar for both groups (median pre-mRS, 0 IQR 0 vs. 0 IQR 0, p = 0.974). PCS patients had a lower mRS at 90 days (median mRS90d 1, IQR 2 vs. 2, IQR 4, *p* = *0.010*) and good outcome, defined as mRS90d ≤ 2, was more frequent among PCS patients (76.3% vs. 62.9%, *p* = *0.022*). There was no difference in the duration of hospital stay or in the occurrence of complications within the 90 days follow-up period between the two stroke territories (Table 3).

**Table 3:** Patient characteristics—scores and course of disorder.

	All n = 517 (%)	ACS n = 437 (%)	PCS n = 80 (%)	P value
**Vital parameters on admission, median (IQR)**				
Systolic BP, mmHg	153 (31)	154 (32)	151 (39)	0.449
Diastolic BP, mmHg	85 (20)	86 (21)	82 (17)	0.169
Glucose, mmol/l	6.4 (1.7) n = 514	6.3 (1.7) n = 435	6.6 (2.0) n = 79	0.292
Creatinine, µmol/l	79 (27) n = 516	78 (27) n = 436	82.5 (26) n = 80	0.199
**Clinical scores, median (IQR)**				
NIHSS on admission	9 (11) n = 513	10 (11) n = 436	5 (8) n = 77	** < 0.001**
NIHSS at 24 h	4 (9) n = 468	5 (9) n = 402	2 (5) n = 66	** < 0.001**
Pre-mRS	0 (0)	0 (0)	0 (0)	0.974
mRS at 90d	1 (3)	2 (4)	1 (2)	**0.010**
Good outcome (mRS90d ≤ 2), n (%)	336 (65.0)	275 (62.9)	61 (76.3)	**0.022**
**Hospital stay, median (IQR)**				
Hospital stay, days	8.93 (8) n = 486	8.97 (8) n = 410	8.89 (7) n = 76	0.708
**Complications within 90 days, n (%)**				
Recurrent stroke	6 (1.2)	6 (1.4)	0 (0)	0.597
Symptomatic ICH	13 (2.5)	13 (3.0)	0 (0)	0.236
Death in Hospital	31 (6.0)	27 (6.2)	4 (5.0)	1.000

Scores and course of disorder of all 517 patients and patient groups according to stroke territory, ACS and PCS. Data are expressed as number of patients (n) and percentages or median and interquartile range (IQR). BP indicates blood pressure; *NIHSS,* National Institutes of Health Stroke Scale; *mRS,* modified Rankin Scale; *ICH,* intracranial haemorrhage. P values were obtained according to Mann–Whitney U test, Pearson’s Chi-squared test and Fisher’s Exact test, where appropriate. P values < 0.05 are shown in bold.

Vascular results including occlusion or stenosis in the suspected ischaemic territory did not differ between PCS and ACS (Table 4).

**Table 4:** Patient characteristics—imaging.

	All n = 517 (%)	ACS n = 437 (%)	PCS n = 80 (%)	P value
**First Imaging, n (%)**				0.702
CT	434 (83.9)	368 (84.2)	66 (82.5)	
MRI	83 (16.1)	69 (15.8)	14 (17.5)	
**Vascular result, n (%)**				0.090
Occlusion in suspected ischaemic territory	292 (57.0) n = 512	255 (58.8) n = 434	37 (47.4) n = 78	
Stenosis 50–99% in suspected ischaemic territory	81 (15.8) n = 512	67 (15.4) n = 434	14 (17.9) n = 78	
No abnormality	139 (27.1) n = 512	112 (25.8) n = 434	27 (34.6) n = 78	

Imaging characteristics of all 517 patients and patient groups according to stroke territory, ACS and PCS. Data are expressed as number of patients (n) and percentages. CT indicates computer tomography; *MRI,* magnetic resonance imaging; *PWI,* perfusion weighted imaging. P values were obtained according to Pearson’s Chi-squared test and Fisher’s Exact test, where appropriate. P values < 0.05 are shown in bold.

### Predictors for posterior versus anterior circulation stroke territory

We investigated the influence of age, sex, diabetes, atrial fibrillation, hypertension and prior stroke or TIA on the stroke territory to seek out potential predictors for PCS versus ACS. The factors were selected due to the observed differences in their occurrence in PCS vs. ACS in previous studies, their overall frequency in the population and their prognostic impact. In the multivariate binary logistic regression analysis, both diabetes (OR 0.349, 95% CI 0.122–0.994, *p* = *0.049*) and atrial fibrillation (OR 0.496, 95% CI 0.263–0.936, *p* = *0.030*) made PCS less likely than ACS (Table 5).

**Table 5:** Binary logistic regression analysis—predictors for PCS vs. ACS territory.

	Odds ratio	95% CI	P value
**Stepwise multivariate binary logistic regression analysis**			
MH diabetes	0.349	0.122–0.994	**0.049**
MH atrial fibrillation	0.496	0.263–0.936	**0.030**

Multivariate stepwise backward binary logistic regression analysis of predictors for stroke in the posterior compared to the anterior cerebral circulation (PCS vs. ACS). The influence of age, sex, diabetes, atrial fibrillation, hypertension and prior stroke or TIA on the stroke territory was investigated. Data are expressed as odds ratio and the 95% CI of the odds ratio. 95% CI indicates 95% confidence interval; *MH,* medical history. P values < 0.05 are shown in bold.

### Predictors for good functional outcome in posterior and anterior circulation stroke

Age, sex, medical history of diabetes, atrial fibrillation, hypertension, dyslipidaemia, smoking, previous stroke or TIA, the admission NIHSS, knowledge of symptom onset and the onset-to-treatment time were examined for their predictive impact on good functional outcome defined as ≤ 2 on the mRS at 90 days after stroke, for each stroke territory respectively.

In the multivariate binary logistic regression analysis, the admission NIHSS remained the only predictor for functional outcome in PCS with a higher NIHSS making a good functional outcome less likely (OR 0.864, 95% CI 0.790–0.944, *p* = *0.001*). For ACS, the multivariate binary logistic regression analysis showed that an increase in age (OR 0.952, 95% CI 0.935–0.970, *p* < *0.001*) and an increase in the admission NIHSS (OR 0.840, 95% CI 0.806–0.876, *p* = *0.000*) made a good functional outcome less likely. On the other hand, a known symptom onset was associated with a good functional outcome (OR 1.869, 95% CI 1.111–3.144, *p* = *0.018*) (Table 6).

**Table 6:** Binary logistic regression analysis—predictors for good outcome in PCS and ACS.

	Odds ratio	95% CI	P value
**Stepwise multivariate binary logistic regression analysis**			
PCS			
Admission NIHSS	0.864	0.790–0.944	**0.001**
ACS			
Age	0.952	0.935–0.970	** < 0.001**
Admission NIHSS	0.840	0.806–0.876	** < 0.001**
Known symptom onset	1.869	1.111–3.144	**0.018**

Multivariate stepwise backward binary logistic regression analysis for outcome in posterior circulation stroke (PCS) and anterior cerebral circulation stroke (ACS), separately. Age, sex, medical history of diabetes, atrial fibrillation, hypertension, dyslipidaemia, smoking, previous stroke or TIA, the admission NIHSS, knowledge of symptom onset and the onset-to-treatment time were examined for their predictive impact on good functional outcome defined as ≤ 2 on the mRS at 90 days. Data are expressed as odds ratios and the 95% CI of the odds ratios. 95% CI indicated 95% confidence interval; *NIHSS,* National Institutes of Health Stroke Scale. P values < 0.05 are shown in bold.

## Discussion

Stroke location and affected vascular territory crucially determine clinical symptoms. However, in addition to these anatomical differences, our results support the hypothesis of different patient baseline characteristics for PCS versus ACS, even for acute stroke patients all selected for thrombolysis and/or thrombectomy. In our descriptive analysis, we found differences in patients with PCS versus ACS. Multivariate logistic regression analyses revealed that the presence of diabetes and atrial fibrillation made an ACS more likely than a PCS. Furthermore, specific predictors of favourable outcome were different in ACS (younger age, lower admission NIHSS, known symptom onset) and PCS (lower admission NIHSS).

In our cohort of patients with ischaemic stroke receiving acute stroke therapy, 15.5% of strokes occurred in the posterior cerebral circulation. This proportion is similar to observations in treatment-only cohorts^13,15,22^. PCS patients in our cohort were more often male, less often suffering from diabetes or atrial fibrillation, had lower admission NIHSS scores compared to ACS and less often underwent mechanical thrombectomy. Moreover, we observed a better three months functional outcome after acute stroke therapy in PCS. Our findings are in line with a recent data analysis from the SITS-MOST (Safe Implementation of Thrombolysis in Stroke Monitoring Study) registry, which had intracerebral haemorrhage (ICH) after intravenous thrombolysis as primary outcome, and found less ICH in patients with PCS strokes, along with a higher proportion of patients reaching functional independence (mRS 0–2) at 3 months^16^.

We could not confirm the observation of PCS patients being younger than ACS patients^8,13,15,26^. Similar to others, we found more men among patients suffering a PCS^5,8,9,15,26,27^. The reason for this sex discrepancy is unclear. Vavilala et al.’s findings of a lower autoregulation capacity in the basilar artery in teenage boys compared to girls might to some extent offer an explanation of the male preponderance in posterior circulation stroke^28^. Atrial fibrillation was previously found more frequently in patients with ACS^5,8,9,15^. On the other hand, the lower incidence of diabetes in PCS contrasts with previous observations which indicated an equal prevalence of diabetes in PCS and ACS^5,13,15,21,22,26,27^ or even a higher prevalence in PCS patients^7–9,29^. While we did not find any differences between PCS and ACS patients concerning pre-stroke medication, other studies suggest a greater use of antihypertensives^5^ and platelet inhibitors in ACS^10,22^. In our descriptive analysis, there were no statistically significant differences in stroke aetiology between the two stroke territories. Previous studies detected a higher incidence of cardiac stroke in ACS and small artery disease in PCS^5,22,27^. These discrepancies are likely due to different patient cohorts, since we considered only patients receiving acute stroke treatments, thus excluding very mild strokes.

The greater frequency of thrombectomy in ACS patients was expected. IAT is safe and effective in PCS and in ACS^14^. The longer onset to treatment time (OTT) in PCS patients with known symptom onset who received thrombolysis was related to the onset-to-door (ODT) rather than the door-to-treatment time (DTT). Several studies observed longer OTT in PCS compared to ACS receiving IVT^14,15,22,26^ and elongated ODT in PCS regardless of acute treatment or knowledge of symptom onset^5,30^. Possible explanations for the pre-hospital time delay in PCS are less obvious stroke symptoms (such as vertigo and double vision compared to hemiplegia or aphasia) and less severe neurological symptoms as judged from the lower admission NIHSS in PCS. However, even though a subgroup of PCS patients arrived later at the hospital, subsequently treated with longer OTT, there was no disadvantage regarding functional outcome at three months detectable in our analysis.

In line with our analysis, the admission NIHSS is often lower in PCS^5,9,11,13,21,22,26,27^, since this score is designed to assess ACS-induced deficits^18^. In our cohort, the pre-stroke mRS, which describes functional impairment, was equally low in PCS and ACS. Interestingly, the mRS after 90 days was lower in PCS, and functional independence defined as mRS ≤ 2 after 90 days was significantly more frequent in PCS patients (76.3% vs. 62.9%, *p* = *0.010*). Complications within three months follow-up including recurrent strokes and symptomatic intracranial haemorrhage (ICH) occurred with similar frequencies in PCS and ACS.

We confirmed differences in patient characteristics and treatment variables between ACS and PCS. However, one major goal of our study was to explore variables that would favour the occurrence of ACS versus PCS, and subsequently, to find variables that would indicate 3-months disability as a measure of treatment success for either territory.

Some studies have reported that atrial fibrillation occurs more frequently in ACS, especially in women above the age of 80 years^31^, while the evidence regarding diabetes as well as further predictors for stroke territory—such as sex, cardiovascular risk factors and stroke mechanism—is conflicting^5,9^. We found that both diabetes and atrial fibrillation favoured the presence of ACS to PCS. There was no evidence that age, sex, hypertension and prior stroke or TIA had an effect on the presence of ACS vs. PCS.

In addition, predictors for 3-months disability were different for patients suffering ACS vs. PCS. The NIHSS on admission was the only predictor for functional outcome in PCS. In contrast, in ACS, in addition to the admission NIHSS, known symptom onset and age predicted outcome after stroke and acute stroke therapy. The correlation of the admission NIHSS with outcome in acutely treated patients has been described before for PCS^14,32^ as well as for ACS patients^21,33^. Many other factors have been proposed to influence outcome after stroke, but either no distinction was made between stroke territories^13,24,29^ or acutely treated and untreated patients were investigated as one entity^8^. In our analysis, we additionally considered sex, medical history of diabetes, hypertension, dyslipidaemia, smoking, previous stroke or TIA and onset to treatment time, which, however, showed no significant effect on functional outcome neither in ACS nor in PCS patients.

It is interesting that the admission NIHSS independently predicted outcome in PCS, although its validity for the assessment of stroke severity in PCS has been questioned^34^. We only considered patients with acute stroke treatment in our analysis. Therefore, the median admission NIHSS score may have been higher compared to non-treated or mixed cohorts, which could have influenced its predictive value for PCS patients. A known symptom onset facilitates a faster identification of stroke symptoms and prompt therapeutic interventions, ideally resulting in a better functional outcome. However, the OTT did not prove to be an independent outcome predictor in either PCS or ACS. For ACS, knowledge on symptom onset may have outweighed the influence of the OTT on the mRS in multivariate regression analysis. For PCS, the OTT has been shown to be less crucial in thrombolysis^35^ which might explain the lack of influence of known symptom onset and OTT on functional outcome in PCS patients.

Our findings imply that differences in patient characteristics between PCS and ACS exist in stroke patients that qualify for acute stroke treatment. Fortunately, the familiar issue of elongated time intervals in the workup of PCS was not very pronounced in our analysis. Although the NIHSS might not entirely capture stroke severity in PCS, its assessment might have prognostic value. Against our expectations and in contrast to Dornak et al.’s findings of a negative correlation of age and outcome in thrombolysed PCS patients^15^, in our analysis older age indicated worse outcome in ACS patients, while age did not correlate with functional outcome in PCS. While an existing influence could be concealed by the limited number of patients, this observation should be considered in assessing the eligibility for acute stroke treatment for PCS. In our analysis, we only considered ischaemic stroke patients receiving acute stroke treatment to evaluate outcome of these treatments in patients with PCS versus ACS. We did not compare treated with untreated patient groups, as our goal was not to reaffirm the efficacy of thrombolysis and thrombectomy in ACS and PCS^12–16^.

One limitation of this analysis is its relatively small number of patients, especially in the subgroup of PCS. This might have contributed to the fact that less outcome predictors were identified in multivariate regression analysis for PCS compared to ACS. In addition, our analysis only included patients from a single centre and the data analysis was performed retrospectively. The strength of our analysis lies in the large number of variables that were compared between the two patient groups, including pre- and post-stroke as well as treatment-specific factors.

Overall, our analysis of acutely treated stroke patients supports the hypothesis of different patient profiles for posterior versus anterior circulation strokes. We identified predictors for the stroke territory as well as predictors for functional outcome for each vascular territory respectively. Knowledge about specific characteristics of patients with ACS and PCS including outcome predictors of thrombolysis and thrombectomy in these patients may aid in improving treatment decisions in acute stroke.

## Methods

### Patients

This retrospective data analysis contains patient data from the Swiss Stroke Registry of the University Hospital Zurich (USZ) from 2014 until the end of 2017. The Swiss Stroke Registry enlists every patient presenting with suspected stroke (< 7 days before admission) at the USZ. Data were collected by the treating physician. Stroke treatments at the USZ Stroke Center were performed according to the current guidelines of the European Stroke Organization^36^. Accordingly, intravenous thrombolysis was performed within 4.5 h of symptom onset, and thrombectomy within 6 h of symptom onset in patients without contraindications. In some patients, treatment windows were extended based on advanced imaging such as small core and/or mismatch. All data was reviewed from the electronic patient files of the clinical information system and corrected if missing or inconclusive. Inclusion criteria were ischaemic stroke and acute stroke therapy with intravenous thrombolysis or intra-arterial therapy, i.e. mechanical thrombectomy. Haemorrhagic strokes and transient ischaemic attacks were excluded. Patients with onset-to-treatment times greater 24 h, in-hospital events and patients with missing information on three months functional outcome were excluded. Consent to research was obtained as written informed consent from all participants and/or their legal guardians or waived according to the ethics protocol KEH-ZH-Nr. 2014-0304 “Bildgebungsprädikatoren für die Erholung nach Schlaganfall (PREDICT)”, approved by the ethics committee Kantonale Ethikkommission Kanton Zürich (KEK ZH); and all methods were carried out in accordance with relevant guidelines and regulations.

Patients were divided into two groups according to their stroke territory in posterior (PCS) versus anterior circulation ischaemic stroke (ACS) by clinical and radiological findings. ACS was defined as a stroke in the territory supplied by the anterior choroidal arteries, the medial cerebral arteries and the anterior cerebral arteries together with their supplying vascular territory. PCS was likewise defined as a stroke in the vascular territory of the vertebral arteries, the basilar artery and the posterior cerebral arteries. To obtain homogenous comparable groups, patients with concurrent posterior and anterior circulation strokes were excluded. All patients received acute stroke therapy by either intravenous thrombolysis (IVT) or intra-arterial therapy (IAT) or a combination of both treatments. Stroke severity was assessed by the National Institutes of Health Stroke Scale (NIHSS) which captures the degree of neurological deficits in acute ischaemic stroke patients, ranging from 0—no neurological deficits—to a total of 42 points^37^. In the Swiss Stroke Registry, the NIHSS is assessed on admission and after 24 h. Both pre-stroke disability and functional outcome were quantified by the modified Rankin Scale (mRS)^38^ which assesses functional independence, ranging from 0—no constraint—to 6—death. In the Swiss Stroke Registry, the mRS is assessed retrospectively on admission and three months after stroke. A mRS ≤ 2 represents functional independence. Stroke aetiology was assessed according to ASTRAL, the Acute STroke Registry and Analysis of Lausanne^5^, which is based on the TOAST classification^39^.

### Statistical analyses

Statistical analyses were performed using IBM SPSS Statistics 25. The two patient groups ACS and PCS were compared regarding demographic and clinical characteristics. Continuous variables are shown as median and interquartile range. Nominal variables are expressed as fractions and percentages. The Mann–Whitney U test for two independent samples was performed to compare continuous variables between the two groups. The Chi-squared test and the Fisher’s Exact test (for expected values less than five) were conducted to compare nominal variables. Binary logistic regression analyses were performed including variables in a stepwise backward manner to identify predictors for posterior versus anterior circulation stroke territory and to investigate predictors for functional outcome (mRS 90d) for each stroke territory. The mRS was dichotomized and good outcome was defined as a score of ≤ 2 on the mRS at 90 days. Results are expressed as odds ratios and the 95% confidence interval (CI) of the odds ratios. A two-sided significance level of alpha = 0.05 was considered. Data are shown in tables.

## Data Availability

All data generated or analysed during this study are included in this published article. The datasets generated during and/or analysed during the current study are available in anonymized form from the corresponding author upon reasonable request.
